# Metabolomics Analysis Reveals Characteristic Functional Components in Pigeon Eggs

**DOI:** 10.3390/metabo15020122

**Published:** 2025-02-12

**Authors:** Rui Zhang, Lingling Chang, Xinyue Shen, Qingping Tang, Chunyu Mu, Shengyong Fu, Zhu Bu

**Affiliations:** Jiangsu Institute of Poultry Science, Yangzhou 225100, China; zrjindy@163.com (R.Z.); jqscll@163.com (L.C.); shenxy0618@163.com (X.S.); tqp0979@163.com (Q.T.); muchunyu521@126.com (C.M.); xnfsy@163.com (S.F.)

**Keywords:** pigeon egg, chicken egg, quail egg, metabolomics

## Abstract

We aimed to identify the characteristic functional components of pigeon eggs and the differences among pigeon, chicken, and quail eggs. We analyzed the metabolite profiles of three kinds of eggs using an untargeted metabolomics-based approach to better understand the differences in metabolites among pigeon, chicken, and quail eggs. Then, we quantitatively validated the differences in abundance of partial metabolites through a targeted metabolomics-based approach. A total of 692 metabolites were identified in the three types of eggs. A total of 263 significantly differentially abundant metabolites were found between pigeon eggs and chicken eggs, and 263 significantly differentially abundant metabolites were found between pigeon eggs and quail eggs. The metabolites that were significantly more abundant in pigeon eggs than in other eggs were mainly lipids, lipid-like molecules, nucleosides, nucleotides, and their analogues. We identified the eight metabolites that were significantly greater in abundance in pigeon eggs than in chicken eggs and quail eggs and quantitatively validated the differences in abundance of these metabolites. Our study demonstrates that there are more functional components in pigeon eggs than chicken eggs and quail eggs, especially for the prevention and treatment of various disordered glucose and lipid metabolism-related diseases. The discovery of these differentially abundant metabolites paves the way for further research on the unique nutritional functions of pigeon eggs and the further utilization of pigeon egg products.

## 1. Introduction

Poultry eggs, as nutritional reservoirs for embryo development, are recognized as important dietary sources for humans and can be used in pharmacological preparations [1]. Eggs are rich in protein and fatty acids, almost up to 98% of the egg protein can be absorbed effectively by the human body [2], and the associated fat-soluble compounds and the type and ratios of fatty acids are an important determinant of human health [3]. Iodine and vitamin D deficiencies have been observed in the world. Eggs are rich in iodine and vitamin D, suggesting that eggs, including processed forms, can be an important dietary source of iodine and vitamin D [4]. Eggs contain not only nutrients but also a number of regulatory and defense protein factors to ensure proper development and to protect against bacterial and viral infections. These substances can be of great importance for humans and can find applications as pharmacological preparations [5]. An example is vitellogenin, which, due to being a precursor for yolkin, a newly discovered polypeptide complex with the ability to stimulate human blood to produce cytokines, plays a role via functioning as immunomodulating molecules [6].

While chicken eggs are the most commonly consumed poultry eggs, eggs from various poultry species are commercially available in different areas of the world. Due to divergent evolutionary processes, eggs from different poultry species exhibit unique properties, including the morphometric traits and unique eggshell patterns of the eggs [7,8]. The eggs of different species contain different nutritional components, including proteins [9], lipids [10,11], trace elements [12], and so on [1]. Compared to the pigeon, there is a closer evolutionary relationship between the chicken and the quail. The gel of pigeon albumen is transparent, while the albumens of other species are turbid gel [9]. Color difference is the most intuitive indicator for food; hence, transparent pigeon eggs may be more attractive to consumers. In addition, there are some unique proteins that only exist in pigeon albumen, such as haptoglobin [13]. Unlike the egg white counterparts of chicken and quail, pigeon egg white glycoproteins contain terminal Gala1-4Gal [14]. Food allergy is an immune-mediated hypersensitivity to specific proteins in food. Ovomucoid in chicken eggs is one of the main causes of allergy reactions, which is an IgE-mediated food allergy [15]. Through skin prick tests in children with hen’s egg allergy (HEA), it is found that the sensitization is the least frequent in pigeon eggs, compared to chicken eggs and quail eggs [16].

In China, chicken eggs and quail eggs are inexpensive and plentiful, and duck eggs are usually processed into salted eggs and preserved eggs; however, pigeon eggs are less abundant but expensive, usually considered high-grade consumer products. Pigeon eggs are also known as “animal ginseng” and are used as part of a medicated diet in individuals of all ages [17]. In recent years, with the upgrading of people’s consumption of pigeon eggs, research on the nutritional value of the eggs has gradually begun. The differences in conventional nutritional components in pigeon eggs, such as protein, amino acids, vitamins, fatty acids, cholesterol, and trace element, compared to other poultry eggs have been detected by conventional detection methods [9,11,18] and proteomic analysis [13]. Although pigeon eggs are important for Chinese recipes, the functional constituents of pigeon eggs and their dietary effects are not clear. In addition, counterfeit eggs, such as cheap chicken eggs and quail eggs being sold as pigeon eggs, can be found on the market. Therefore, it is particularly important to identify the specific functional components of pigeon eggs and the differences among pigeon, chicken, and quail eggs. Metabolomics is an emerging technology, and it allows the study of multiple metabolites present in a cell, a tissue, or an organism and can be used to identify differences between foods [19,20]. Untargeted metabolomics is a systematic and unbiased method for studying the composition and changes in small molecule metabolites in organisms. Untargeted metabolomics based on high-resolution mass spectrometry has become a powerful tool for analyzing natural components in various food materials and processed products [21]. This method has been used to explore the effects of different varieties, processing methods, storage times, and other factors on food quality [22,23,24]. Therefore, we used UHPLC-MS/MS to identify differentially abundant metabolite detection in pigeon, chicken, and quail eggs with the aim of providing insightful information on how the species affects the egg composition.

## 2. Materials and Methods

### 2.1. Sample Collection

Fresh eggs of pigeons, chickens, and quails were collected randomly from the Jiangsu Institute of Poultry Science within 24 h after being laid and utilized in this study. The eggs of different poultry were collected in April 2024 at 2:00 pm every day. We prefabricated the whole egg mixture and placed it in liquid nitrogen for quick freezing, then transferred it to −80 °C for storage. The composition of the diet is shown in Table 1.

### 2.2. Metabolites Extraction for Untargeted Metabolomics Analysis

Six pigeon eggs (P), six chicken eggs (C), and six quail eggs (Q) were selected randomly. The whole egg liquid (100 mg per sample) was mixed evenly, and the homogenate was resuspended with prechilled 80% methanol by well vortex. The samples were incubated on ice for 5 min and then were centrifuged at 15,000× *g* and 4 °C for 20 min. Some of the supernatant was diluted to a final concentration containing 53% methanol using LC-MS-grade water. The samples were subsequently transferred to a fresh Eppendorf tube and then were centrifuged at 15,000× *g* and 4 °C for 20 min. Finally, the supernatant was injected into the LC-MS/MS system for analysis. In order to control the quality of the experiment, equal volumes of samples were taken from each experimental sample and mixed well as quality control (QC) samples. A 53% methanol aqueous solution was set as a blank sample for removing background ions.

### 2.3. UHPLC-MS/MS for Untargeted Metabolomics Analysis

UHPLC-MS/MS analysis was performed using a Vanquish UHPLC system (Thermo Fisher, Hannover, Germany) coupled with an Orbitrap Q Exactive^TM^ HF mass spectrometer (Thermo Fisher, Hannover, Germany) or Orbitrap Q Exactive^TM^ HF-X mass spectrometer (Thermo Fisher, Hannover, Germany) at Novogene Co., Ltd. (Beijing, China). Samples were injected onto a Hypersil Gold column (100 × 2.1 mm, 1.9 μm) using a 12 min linear gradient at a flow rate of 0.2 mL/min. The eluents for the positive and negative polarity modes were eluent A (0.1% FA in Water) and eluent B (Methanol). The solvent gradient was set as follows: 2% B, 1.5 min; 2–85% B, 3 min; 85–100% B, 10 min; 100–2% B, 10.1 min; and 2% B, 12 min. The Q Exactive^TM^ HF-X mass spectrometer was operated in positive/negative polarity mode, with a spray voltage of 3.5 kV, capillary temperature of 320 °C, sheath gas flow rate of 35 psi, aux gas flow rate of 10 L/min, S-lens RF level of 60, and aux gas heater temperature of 350 °C.

### 2.4. Identification and Relative Quantification of Metabolites

The raw data files generated by UHPLC-MS/MS were processed using Compound Discoverer 3.3 (CD3.3, Thermo Fisher) to perform peak alignment, peak picking, and quantitation for each metabolite. After that, peak intensities were normalized to the total spectral intensity. The normalized data were used to predict the molecular formula based on additive ions, molecular ion peaks, and fragment ions. Then, peaks were matched with the mzCloud (https://www.mzcloud.org/ (accessed on 11 May 2024)), mzVault, and MassList databases to obtain accurate qualitative and relative quantitative results.

### 2.5. Metabolites Extraction for Targeted Metabolomics Analysis

Eight pigeon eggs and eight chicken eggs were selected randomly. Two eggs were mixed into a sample. To extract metabolites from samples, 400 μL of cold methanol/acetonitrile (1:1, *v*/*v*) extraction solvent was added to remove the protein and extract the metabolites and then adequately vortexed. For absolute quantification of the metabolites, stock solutions of stable isotope internal standards were added to the extraction solvent simultaneously. The mixture was collected into a new centrifuge tube and centrifuged at 14,000× *g* for 20 min at 4 °C to collect the supernatant.

### 2.6. HPLC-MS/MS Analysis for Targeted Metabolomics Analysis

Analyses were performed using a UHPLC (1290 Infinity LC, Agilent Technologies, Santa Clara, CA, USA) coupled to a QTRAP MS (6500+, AB Sciex, Framingham, MA, USA) at Shanghai Applied Protein Technology Co., Ltd. (Shanghai, China). The analytes were separated on HILIC (Waters UPLC BEH Amide column, 2.1 mm × 100 mm, 1.7 µm) and C18 columns (Waters UPLC BEH C18-2.1 × 100 mm, 1.7 μm). For HILIC separation, the column temperature was set at 35 °C, and the injection volume was 2 μL. Mobile phase A included 90% H_2_O + 2 mM ammonium formate + 10% acetonitrile, and mobile phase B included 0.4% formic acid in acetonitrile. A gradient (85% B at 0–1 min, 80% B at 3–4 min, 70% B at 6 min, 50% B at 10–15.5 min, and 85% B at 15.6–23 min) was then initiated at a flow rate of 300 μL/min. For RPLC separation, the column temperature was set at 40 °C, and the injection volume was 2 μL. Mobile phase A included 5 mM ammonium acetate in water, and mobile phase B included 99.5% acetonitrile. A gradient (5% B at 0 min, 60% B at 5 min, 100% B at 11–13 min, and 5% B at 13.1–16 min) was then initiated at a flow rate of 400 μL/min. The sample was set at 4 °C during the whole analysis process.

The 6500+ QTRAP (AB SCIEX) was performed in positive and negative switch modes. The ESI-positive source conditions were as follows: source temperature: 580 °C; Ion Source Gas1 (GS1): 45; Ion Source Gas2 (GS2): 60; curtain gas (CUR): 35; and IonSpray Voltage (IS): +4500 V. The ESI-negative source conditions were as follows: source temperature: 580 °C; Ion Source Gas1 (GS1): 45; Ion Source Gas2 (GS2): 60; curtain gas (CUR): 35; and IonSpray Voltage (IS): −4500 V. The MRM method was used for mass spectrometry quantitative data acquisition. Polled QC samples were set in the sample queue to evaluate the stability and repeatability of the system.

### 2.7. Data Analysis

These metabolites obtained through untargeted metabolomics were annotated using the KEGG database (https://www.genome.jp/kegg/pathway.html (accessed on 11 May 2024)), HMDB database (https://hmdb.ca/metabolites (accessed on 11 May 2024)), and LIPID Maps database (http://www.lipidmaps.org/ (accessed on 11 May 2024)). Principal components analysis (PCA) and partial least squares discriminant analysis (PLS-DA) were performed at metaX. The metabolites with variable importance in projection (VIP) > 1, *p*-value < 0.05, and fold change (FC) ≥ 2 or FC ≤ 0.5 were considered to be differential metabolites. Student’s *t*-test was applied to determine the significant differences in the metabolites between pigeon eggs and chicken eggs. *p*-values < 0.05 were used to screen significantly changed metabolites.

## 3. Results

### 3.1. Metabolic Profiling

**Figure 1:**
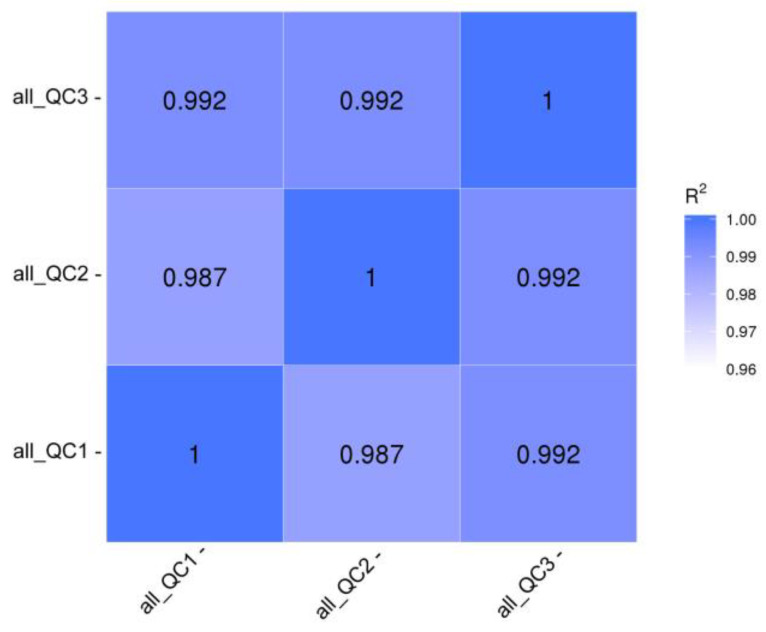
Correlation analysis of QC samples.

**Figure 2:**
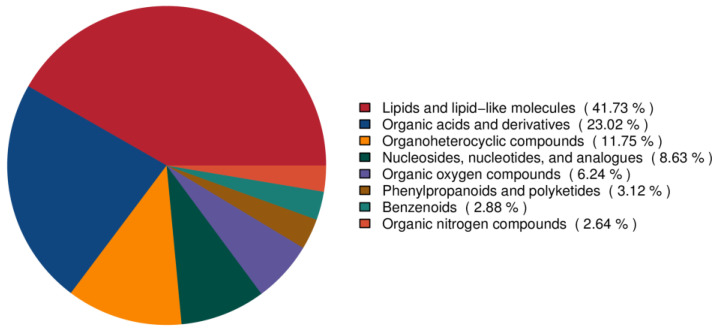
Classification of the metabolites in the three types of eggs.

**Figure 3:**
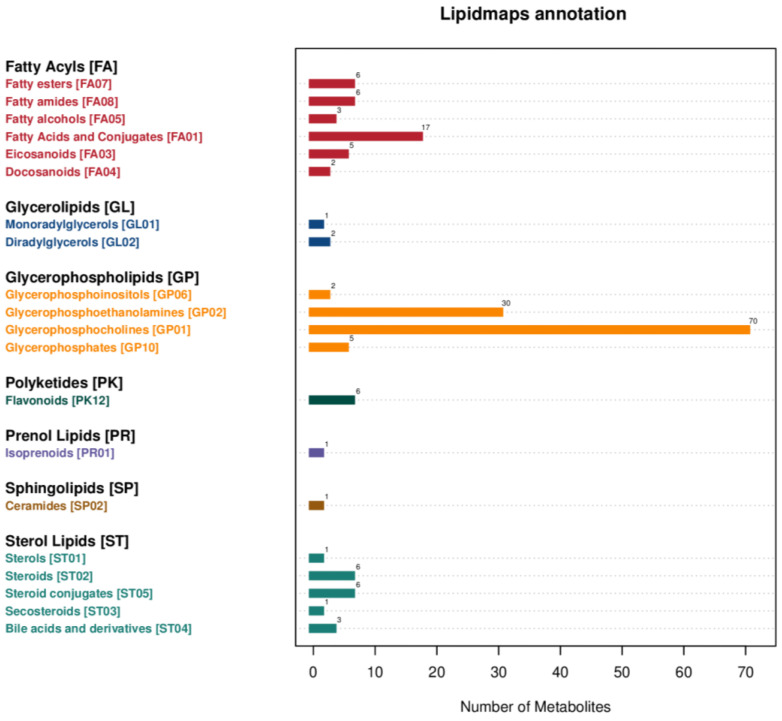
Classification annotation diagram of lipid metabolites identified in the three types of eggs using LIPID MAPS.

Untargeted metabolomics analysis was performed to obtain metabolic profiles of pigeon eggs, chicken eggs, and quail eggs via UHPLC–MS/MS. The metabolites present in each type of egg were determined. The levels of different metabolites were quantitatively analyzed, and Pearson correlation coefficients between the egg samples and QC samples were calculated. The R^2^ values of the QC samples were all greater than 0.98 (Figure 1), indicating good data stability and quality throughout the analysis. A total of 692 metabolites were identified utilizing UHPLC-MS/MS, and 275 of these metabolites could not be annotated; among the 417 metabolites that could be annotated, the predominant metabolites were lipids and lipid-like molecules (41.73%), followed by organic acids and derivatives (23.02%) (Figure 2, Supplementary Table S1). The identified lipid metabolites were classified and annotated using the LIPID MAPS database, and the lipid metabolites were predominantly glycerophospholipids (Figure 3).

### 3.2. Multivariate Statistical Analysis

**Figure 4:**
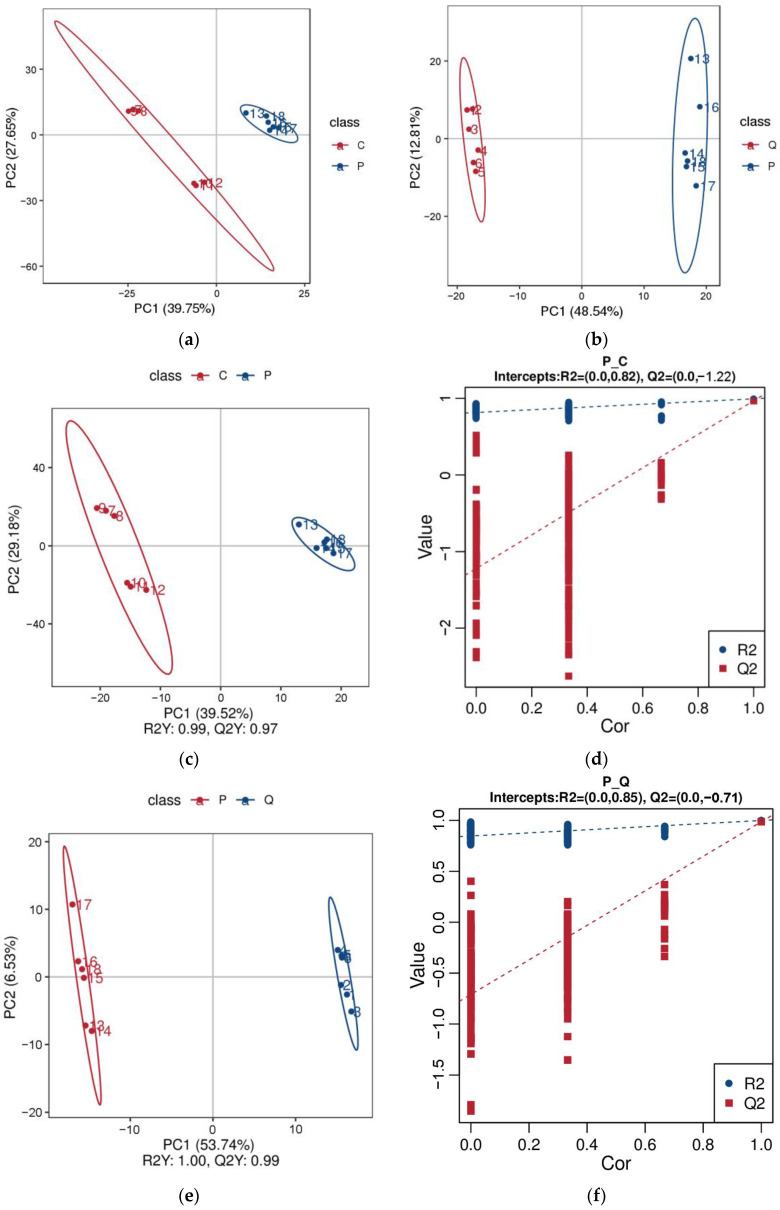
Metabolome quality control analysis. PCA of the metabolites between the P vs. C group (**a**) and the P vs. Q group (**b**). Score scatters from PLS-DA in the P vs. C group (**c**) and the P vs. Q group (**e**). Permutation test of PLS-DA in the P vs. C group (**d**) and the P vs. Q group (**f**).

PCA was used to analyze the overall differences in the abundances of the identified metabolites between samples, and the samples of each egg type were well clustered, indicating good reliability (Figure 4a,b). Pigeon eggs could be clearly distinguished from chicken eggs and quail eggs, indicating that there were differences in metabolite profiles between pigeon eggs and chicken eggs or quail eggs. A PLS-DA model was used to model the relationship between the relative abundances of the different metabolites and sample categories to predict the sample category. The PLS-DA scatter scores and the permutation test confirming the accuracy of the PLS-DA models of the groups are shown in Figure 4c,d. The R2 values were greater than the Q2 value, and the intercept between the Q2 regression line and the Y-axis was less than 0, indicating that the PLS-DA model was sufficiently predictable and was not overfitted. Differentially abundant metabolite screening and analysis were thus carried out.

### 3.3. Identification of Differential Metabolites

**Figure 5:**
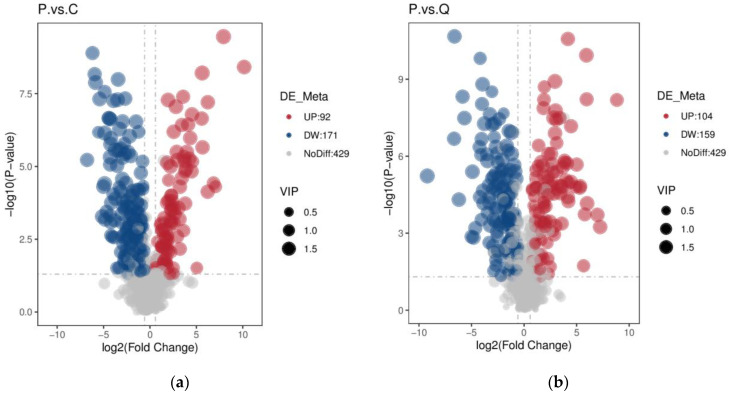
Different metabolites analysis among the different types of eggs. Volcanic plots showing the differential metabolite expression levels between the P vs. C group (**a**) and the P vs. Q group (**b**).

The metabolites with VIP > 1, *p*-value < 0.05, and FC ≥ 2 or FC ≤ 0.5, chosen between different groups, were considered significantly differential metabolites. There were 263 significantly differential metabolites between pigeon eggs (P) and chicken eggs (C) (92 upregulated and 171 downregulated) and 263 significantly differential metabolites between pigeon eggs (P) and quail eggs (Q) (104 upregulated and 159 downregulated) (Figure 5).

**Figure 6:**
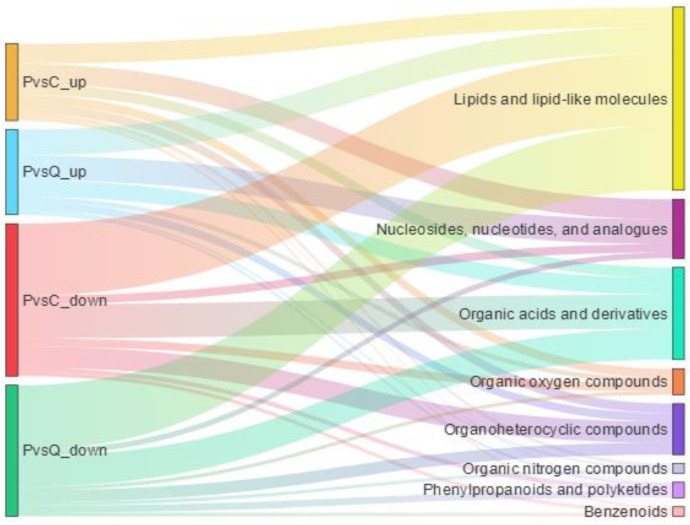
Sankey diagram of the classifications of the differential metabolites among the different types of eggs. PvsC_up: the metabolites significantly more abundant in pigeon eggs than in chicken eggs; PvsQ_up: the metabolites significantly more abundant in pigeon eggs than in quail eggs; PvsC_down: the metabolites significantly less abundant in pigeon eggs than in chicken eggs; and PvsQ_down: the metabolites significantly less abundant in pigeon eggs than in quail eggs.

The classified information of the significantly differential metabolites is shown in Figure 6. The metabolites with significant differences between pigeon eggs and chicken eggs were mainly lipids and lipid-like molecules; nucleosides, nucleotides, and analogues; organic acids and derivatives; and organoheterocyclic compounds. The metabolites with significant differences between pigeon eggs and quail eggs were mainly lipids and lipid-like molecules; nucleosides, nucleotides, and analogues; and organic acids and derivatives.

**Figure 7:**
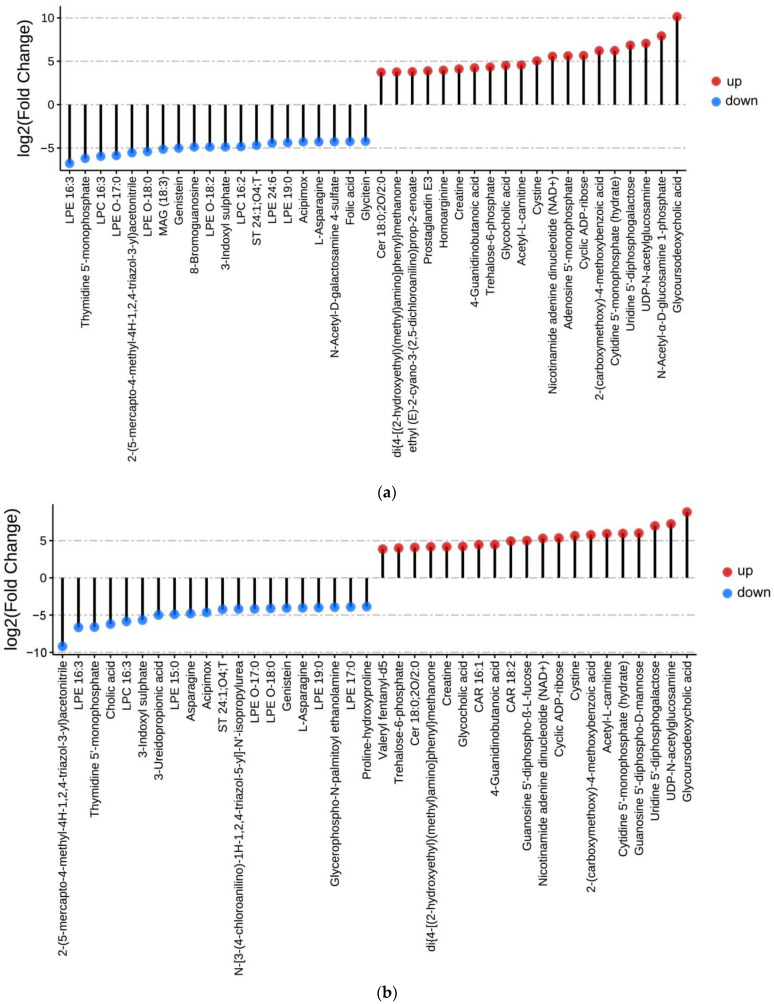
The top 20 up- and downregulated metabolites between each of the two groups. The lollipop diagram showing the top 20 differential metabolites between the P vs. C group (**a**) and P vs. Q group (**b**).

**Figure 8:**
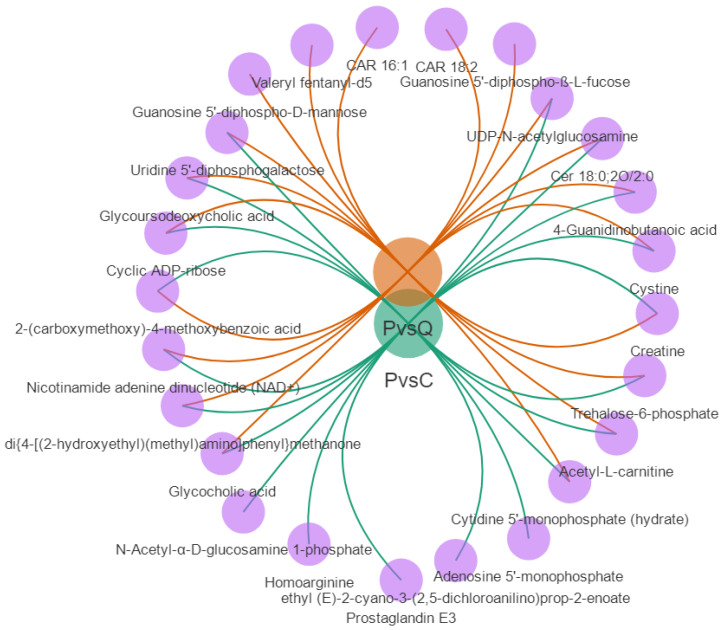
Venn network diagram of differential metabolites. PvsC: the top 20 upregulated metabolites between the P vs. C group; P vs Q: the top 20 upregulated metabolites between the P vs. Q group.

The log-transformed fold-change threshold for significantly differentially abundant metabolites between the two groups was 2, and the top 20 up- and downregulated metabolites between each of the two groups were identified (Figure 7). Among the upregulated metabolites, 15 were significantly upregulated in pigeon eggs compared to both chicken eggs and quail eggs (Figure 8), including glycoursodeoxycholic acid (GUDCA), UDP-N-acetylglucosamine (UDP-GlcNAc), uridine 5′-diphosphogalactose (UDP-Gal), cytidine 5′-monophosphate (5′-CMP), 2-(carboxymethoxy)-4-methoxybenzoic acid, cyclic ADP-ribose (cADPR), nicotinamide adenine dinucleotide (NAD^+^), cystine, acetyl-L-carnitine (ALC), glycocholic acid (GCA), trehalose-6-phosphate (Tre6P), 4-guanidinobutanoic acid (GBA), creatine, di{4-[(2-hydroxyethyl)(methyl)amino]phenyl}methanone, and Cer 18:0;2O/2:0.

### 3.4. Quantitative Validation of the Differential Abundances of Metabolites

**Figure 9:**
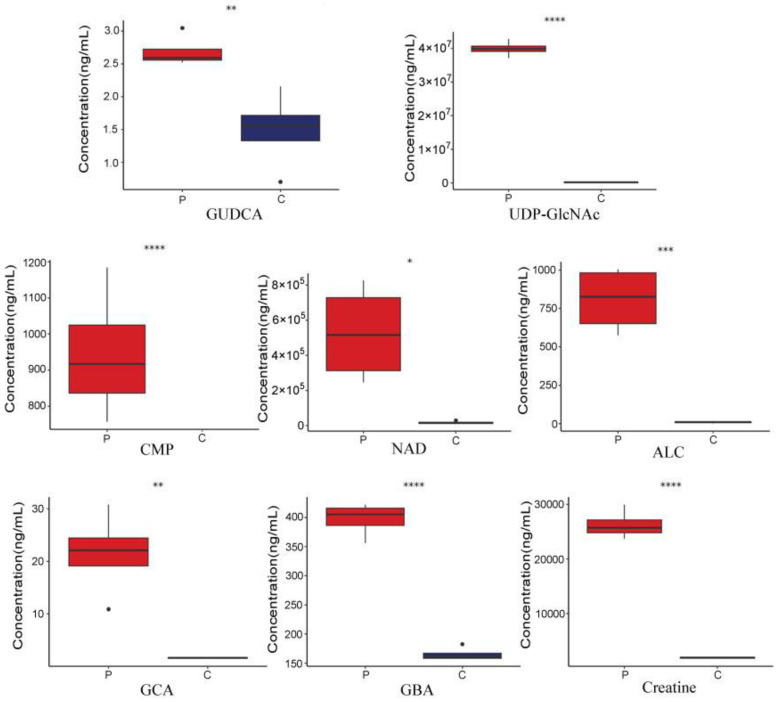
The concentrations of the metabolites in pigeon eggs (P) and chicken eggs (C). The significant difference between groups is represented by the *p*-value, calculated using the *t*-test. **** represents *p* < 0.0001, *** represent *p* < 0.001, ** represent *p* < 0.01, and * represent *p* < 0.05.

UHPLC–MS/MS was used to measure the levels of some of the significantly differentially abundant metabolites, including GUDCA, UDP-GlcNAc, 5′-CMP, NAD^+^, cystine, ALC, GCA, GBA, and creatine. The results are shown in Figure 9, Supplementary Table S2. The differences in the levels of all metabolites, except for cysteine, were consistent with the results of the untargeted metabolomics analysis.

## 4. Discussion

Poultry eggs are among the most important animal products for human nutrition because they contain high-quality proteins, lipids, minerals, and vitamins. As they are the most widely consumed type of poultry egg in the world, most research has focused on the nutritional and functional components of chicken eggs [25]. Eggs are an excellent source of amino acids and vitamin D and contain important nutrients, such as choline and phospholipids, in addition to minerals such as calcium and iron [26]. Quail eggs are gaining popularity because of their unique mottled appearance, inexpensive price, and nutritional density [27]. The yolk of quail eggs has a high protein content and a PUFA/SFA ratio of 0.45, which is considered appropriate for humans [10,28]. In China, pigeon eggs have been recognized as having a variety of medicinal and dietary properties since ancient times and are often used as a high-quality food or in effective postoperative tonics for patients. As pigeon eggs are low in cholesterol, diabetic patients should eat more pigeon eggs if they need to eat eggs [18]. However, research on the characteristic functional components of pigeon eggs is limited. In this study, we aimed to identify the functional components of pigeon eggs using untargeted metabolomics analysis. We identified the top 15 significantly differentially expressed metabolites and validated the levels of 9 of these metabolites by targeted metabolomics analysis. All of these metabolites, except cysteine, were found to be significantly more abundant in pigeon eggs than in chicken and quail eggs. It is noteworthy that the abundances of UDP-GlcNAc and NAD^+^ are very high in pigeon eggs. These results provide support and a theoretical foundation for the development of biomarkers for the pigeon eggs.

UDP-GlcNAc and NAD^+^ have been extensively studied and applied in the fields of medical, health, and beauty. UDP-GlcNAc is a precursor of glycoconjugates, including hyaluronan (HA), and induces protein glycosylation to form O-linked GlcNAc (O-GlcNAcylation). UDP-GlcNAc can modulate the cell microenvironment in many pathologies, including vascular diseases and cancer [29]. As a polysaccharide, HA has high biocompatibility and high water retention, and its main function is to regulate and repair cell growth. It can minimize skin aging and wrinkles by absorbing and retaining moisture, resulting in temporarily round and full skin, widely used in medicine and cosmetics [30,31]. In conjunction with collagen fibers and other macromolecular elements of the extracellular matrix, HA can change the protein stability and gel properties of milk, whey, meat lotion, and fish gelatin [32,33,34,35,36]. The enrichment of this component in pigeon eggs may be related to the formation of the transparent gel of pigeon albumen. NAD^+^ is a nucleoside sugar phosphate linked by two covalent bonds and contains an adenosine monophosphate (AMP) and a nicotinamide mononucleotide (NMN). NAD^+^ is an important coenzyme for hydride-transfer enzymes essential for multiple metabolic processes, including glycolysis, pyruvate dehydrogenase complex activity, the TCA cycle, and oxidative phosphorylation [37]. NAD^+^ can directly and indirectly influence many key cellular functions, including metabolic pathways, DNA repair, chromatin remodeling, cellular senescence, and immune cell function, which are critical for maintaining tissue and metabolic homeostasis and for healthy aging [38]. NAD^+^ homeostasis is important for preventing pellagra, prolonging life, and increasing resistance to infectious diseases and inflammation, and it is important in resisting cardiovascular disease, metabolic syndrome, neurodegenerative diseases, and even cancer [39]. Nicotinic acid, a precursor of NAD^+^, has been added to the poultry diet. However, only pigeon eggs are highly enriched in NAD^+^, which may be caused by species difference.

Some components in pigeon eggs have been used as oral medication for health and disease. GUDCA, a glycine-conjugated form of UDCA, is known to exert neuroprotective effects because of its antiapoptotic, anti-inflammatory, and antioxidant effects [40]. GUDCA supplementation modulates the gut microbiota, increases the abundance of beneficial bacteria, alters bile acid metabolic profiles to some extent [41], improves cholesterol homeostasis, and protects against atherosclerosis progression [42]. GUDCA is associated with glucose control [43], and oral GUDCA supplementation may be of potential translational value in the clinical treatment of type 2 diabetes (T2D) [44]. ALC, an endogenous compound, is the short ester of the L-carnitine isomer that facilitates the transfer of fatty acids from the cytosol to the mitochondria during β-oxidation [45]. ALC has antioxidant, antiapoptotic, and neuroprotective effects [46]. As chronic ALC administration improves energy metabolism in the brain and protects against acute stress exposure [47,48], it shows promise in the treatment of aging and neurodegenerative diseases [49]. Creatine is one of the most popular and widely researched natural supplements. Creatine supplementation has been shown to increase strength and fat-free mass, improve muscle morphology [50], and reduce neuronal cell loss [51]. In addition, 5′-CMP is a nucleoside phosphate that is composed of a ribonucleoside and one phosphate group and is widely applied in the food and pharmaceutical industries [52]. Nucleotides are involved in the metabolism of long-chain polyunsaturated fatty acids and modify the composition of the intestinal microflora and iron absorption in the gut [53]. cADPR, a cyclic derivative of NAD^+^, plays a role in the NAD^+^ signaling pathway as a Ca^2+^-mobilizing second messenger, leading to glucose-induced insulin secretion [54]. There is accumulating evidence for the important role of cADPR in central nervous system (CNS) function [55].

The accumulation of body fat results in disordered glucose and lipid metabolism, which can lead to the development of several cardiovascular diseases, T2D, and other diseases. In recent decades, the prevalence of T2D has increased rapidly in China [56]. Studies have also indicated that alterations in fatty acid and lipid metabolism, important contributors to neuroplasticity, occur in patients with depression [57]. Most of the functional components of pigeon eggs identified in this study are mainly involved in regulating sugar and lipid metabolism, reducing fat deposition, protecting the cardiovascular system and cerebral vessels, nourishing nerves, and creating antioxidative effects. It is not surprising that pigeon eggs may play an important role in the prevention and treatment of various related diseases. These results provide a scientific basis for the dietary therapy theory of pigeon eggs. Pigeon eggs can also be used as a natural source of UDP-GlcNAc and NAD^+^ for the development of beauty and health products. However, whether pigeon eggs actually do these things needs to be verified by further animal experiments.

## 5. Conclusions

In this study, UHPLC–MS/MS-based metabolomics was employed to identify the characteristic functional components of pigeon eggs and the differences among pigeon, chicken, and quail eggs. The metabolites that were significantly more abundant in pigeon eggs than in other eggs were mainly lipids, lipid-like molecules, nucleosides, nucleotides, and their analogues. The top 15 significantly highly expressed metabolites were identified in pigeon eggs, and the levels of 9 of these metabolites were validated by targeted metabolomics analysis. Among them, UDP-GlcNAc and NAD^+^ were the most abundant. The characteristic functional components in pigeon eggs were mainly related to the prevention and treatment of various disordered glucose- and lipid metabolism-related diseases. The discovery of these differentially abundant metabolites paves the way for the development of biomarkers of pigeon eggs and the further utilization of pigeon egg products.

## Figures and Tables

**Table 1:** Ingredients and composition of the diets of different poultry.

	Species	Pigeon	Chicken	Quail
Ingredients				
Maize, %		65.00	54.00	48.00
Soybean meal, %		19.45	22.85	27.85
Wheat, %		10.00	10.00	10.00
Soybean oil, %		0.00	2.50	3.50
Trace elements ^1^, %		0.20	0.20	0.20
Vitamins ^2^, %		0.05	0.05	0.05
Limestone, %		3.00	8.10	8.10
Sodium chloride, %		0.30	0.30	0.30
Dicalcium phosphate, %		2.00	2.00	2.00
Total, %		100.00	100.00	100.00
Nutrition level				
Metabolizable energy, MJ/kg		12.08	11.80	11.90
Protein, %		15.50	16.20	18.00
Calcium, %		1.60	3.50	3.52
Phosphorus (total), %		0.67	0.67	0.68

^1^ The premix provided the following per kg of diets: Fe (as ferrous sulfate) 60 mg, Cu (as copper sulfate) 8 mg, Zn (as zinc sulfate) 66 mg, Mn 65 mg, Se 0.3 mg, and I 1 mg. ^2^ The premix provided the following per kg of diets: VA 12,500 IU, VD 34,125 IU, VE 15 IU, VK 2 mg, thiamine 1 mg, riboflavin 8.5 mg, calcium pantothenate 50 mg, nicotinic acid 32.5 mg, pyridoxine 8 mg, VB 12 5 mg, and biotin 2 mg.

## Data Availability

The data presented in this study are available in Supplementary Materials.

## Supplementary Materials

The following supporting information can be downloaded at: https://www.mdpi.com/article/10.3390/metabo15020122/s1, Table S1: The metabolites of the pigeon egg, chicken egg, and quail egg; Table S2: The abundances of the metabolites in pigeon egg and chicken egg.
